# Mindfulness Training Improves Cognition and Strengthens Intrinsic Connectivity Between the Hippocampus and Posteromedial Cortex in Healthy Older Adults

**DOI:** 10.3389/fnagi.2021.702796

**Published:** 2021-08-27

**Authors:** Gunes Sevinc, Johann Rusche, Bonnie Wong, Tanya Datta, Robert Kaufman, Sarah E. Gutz, Marissa Schneider, Nevyana Todorova, Christian Gaser, Götz Thomalla, Dorene Rentz, Bradford D. Dickerson, Sara W. Lazar

**Affiliations:** ^1^Department of Psychiatry, Massachusetts General Hospital, Harvard Medical School, Boston, MA, United States; ^2^Kopf- und Neurozentrum, Department of Neurology, University Medical Center Hamburg-Eppendorf, Hamburg, Germany; ^3^Department of Neurology, Massachusetts General Hospital, Harvard Medical School, Boston, MA, United States; ^4^Program in Speech and Hearing Bioscience and Technology, Harvard Medical School, Boston, MA, United States; ^5^Department of Behavioral Neuroscience, College of Science, Northeastern University, Boston, MA, United States; ^6^Department of Psychiatry and Neurology, Jena University Hospital, Jena, Germany; ^7^Department of Neurology, Brigham and Women’s Hospital, Harvard Medical School, Boston, MA, United States

**Keywords:** aging, resting state – fMRI, mindfulness, cognitive composite, intervention

## Abstract

Maintaining optimal cognitive functioning throughout the lifespan is a public health priority. Evaluation of cognitive outcomes following interventions to promote and preserve brain structure and function in older adults, and associated neural mechanisms, are therefore of critical importance. In this randomized controlled trial, we examined the behavioral and neural outcomes following mindfulness training (*n* = 72), compared to a cognitive fitness program (*n* = 74) in healthy, cognitively normal, older adults (65–80 years old). To assess cognitive functioning, we used the Preclinical Alzheimer Cognitive Composite (PACC), which combines measures of episodic memory, executive function, and global cognition. We hypothesized that mindfulness training would enhance cognition, increase intrinsic functional connectivity measured with magnetic resonance imaging (MRI) between the hippocampus and posteromedial cortex, as well as promote increased gray matter volume within those regions. Following the 8-week intervention, the mindfulness training group showed improved performance on the PACC, while the control group did not. Furthermore, following mindfulness training, greater improvement on the PACC was associated with a larger increase in intrinsic connectivity within the default mode network, particularly between the right hippocampus and posteromedial cortex and between the left hippocampus and lateral parietal cortex. The cognitive fitness training group did not show such effects. These findings demonstrate that mindfulness training improves cognitive performance in cognitively intact older individuals and strengthens connectivity within the default mode network, which is particularly vulnerable to aging affects.

**Clinical Trial Registration:** [https://clinicaltrials.gov/ct2/show/NCT02628548], identifier [NCT02628548].

## Introduction

As maintaining optimal cognitive functioning throughout the lifespan has become a public health priority, a number of interventions that aim to slow or reverse normal age-related decline have been proposed (Anguera et al., 2013; Rebok et al., 2014; Banducci et al., 2017; Foster et al., 2019). Among those, mindfulness training has been suggested to be an efficacious method for enhancing cognitive functions that decline with age (Gard et al., 2014; Fountain-Zaragoza and Prakash, 2017). Here, in a randomized controlled longitudinal study, we investigated cognitive outcomes and associated neural mechanisms following an 8-week mindfulness meditation- based training program compared to a “brain games” mental training program in cognitively normal older adults. An enhanced understanding of the mechanisms through which these interventions may counteract age-related decline can provide novel insights into training based cognitive improvements and enhance our understanding of neural plasticity in aging.

Current interventions aim to help older adults maintain optimal cognitive functioning either through explicit training regimens that engage specific cognitive functions such as memory (Requena et al., 2016), or use various techniques such as transcranial direct current stimulation (Passow et al., 2017) and neurofeedback (Reis et al., 2016; Jiang et al., 2017). Other interventions aim to improve cognitive capacities indirectly through exercise and diet programs (Colcombe and Kramer, 2003). In addition to limitations associated with their near- and far-transferability (Shipstead et al., 2010; Melby-Lervåg and Hulme, 2016; Grönholm-Nyman et al., 2017), these interventions are also limited in terms of their availability to a broader population of older adults.

More recently, mindfulness training have been proposed as an efficacious intervention to enhance cognitive functions in healthy older adults (Chiesa et al., 2011; Gard et al., 2014; Lao et al., 2016; Fountain-Zaragoza and Prakash, 2017; Cásedas et al., 2020). In line with enhanced attentional performance and preserved gray matter volume in long term meditators (Pagnoni and Cekic, 2007), mindfulness meditation-based interventions have been associated with improvements in attention, memory, executive function, processing speed, as well as general cognition. However, neural mechanisms associated with these improvements have yet to be discovered. Mindfulness meditation emphasizes the skill of meta-awareness to monitor distracting external or internal events such as arising thoughts, in order to maintain attention on the meditative object and prevent the mind from wandering, enhancing meta-cognitive monitoring and meta-cognitive control capacity (Schooler, 2002; Schooler et al., 2011). By targeting these domains through mindfulness training, we hypothesize training-specific, measurable cognitive performance effects through mechanisms that are distinctive from other cognitive training programs that use complex exogenous stimuli to capture and maintain attention (Mozolic et al., 2011).

In the absence of external task-demands, the spontaneous fluctuations in the blood-oxygen-level-dependent signal (BOLD) have been shown to display temporally coherent activity patterns within functional and anatomic systems of the brain (Biswal et al., 1995; Greicius et al., 2003; Seeley et al., 2007). This spontaneous during rest have already been associated with individual variability in human behavior. In older adults, particularly, decreases in cognition have been linked to decreases in intrinsic connectivity of the default network. Neurocognitive aging is associated with reduced deactivation of the default network during task-positive states as well as with decreased within-network connectivity during rest (Ferreira and Busatto, 2013; Madhyastha and Grabowski, 2013; Dennis and Thompson, 2014; Persson et al., 2014; Vidal-Piñeiro et al., 2014). Among default mode network structures, posteromedial cortices that are strongly functionally connected to the medial temporal lobes, are selectively vulnerable to pathology (Sperling et al., 2010). Critically, intrinsic connectivity between these regions, particularly between the posteromedial cortex (PMC) and hippocampus, has been associated with individual differences in memory performance among cognitively intact older individuals (Dickerson and Eichenbaum, 2010; Wang et al., 2010; Ferreira et al., 2016). Morphological investigations of preserved cognitive function in aging corroborate the critical role of these regions in preserving cognitive functioning as well (Good et al., 2001; Bakkour et al., 2013). The rate of cortical thinning in the posteromedial cortex, along with other loci, is strongly associated with the rate of cognitive decline (Dickerson and Wolk, 2012), as well as with progression from mild cognitive impairment to Alzheimer’s dementia (Chételat et al., 2005).

Mindfulness training-related increases in brain structure and function partly overlap with the neural regions implicated in age-related cognitive decline outlined above, in particular the posterior cingulate cortex (PCC) and hippocampus. Morphological investigations of mindfulness training have documented increases in gray matter density in PCC and hippocampus (Hölzel et al., 2011; Wells et al., 2013; Greenberg et al., 2017). Alterations in hippocampal (Engström et al., 2010; Yang et al., 2016) and PCC activity (Hasenkamp and Barsalou, 2012; Garrison et al., 2013; Ellamil et al., 2016), as well as increased connectivity between these regions during both meditation and while resting have also been reported (Brewer et al., 2011; Kilpatrick et al., 2011; Taylor et al., 2013; Wells et al., 2013; Brewer and Garrison, 2014; Garrison et al., 2015; Kral et al., 2019).

Although mindfulness training has been proposed as an efficacious intervention for healthy aging, a mechanistic account of mindfulness training alterations in cognition in older adults is still lacking. Here we aimed to investigate neural mechanisms associated with mindfulness training dependent changes in cognition. To this end, we used a composite test battery that combines measures of episodic memory, executive function, and global cognition, that was developed to track normal age-related cognitive decline as well as to predict early cognitive changes in neurodegenerative diseases (Donohue et al., 2014; Papp et al., 2017). Relying on the overlap in neural regions implicated in age-related cognitive decline and mindfulness training-related changes in neural functioning, we hypothesized an association between increases in cognition and enhanced intrinsic connectivity between the hippocampus and PMC. We specifically hypothesized that a mindfulness-based intervention would improve cognitive function across multiple domains in cognitively normal older adults relative to an active control group, and that these improvements would be associated with: (i) increased intrinsic connectivity between the PMC and hippocampus; and (ii) increased gray matter volumes in these regions.

## Materials and Methods

### Recruitment, Randomization and Blinding

Participants responded to advertising for a “Brain Training Study” and were recruited via a direct mail campaign as well as through various email list-servers. Following completion of all baseline testing as specified below, the randomization module within REDCap was used to randomize participants 1:1 into the two training programs in permuted groups of six, by gender. Study staff conducting subsequent testing visits were blind to group status. Importantly, participants were told that both training programs were effective for promoting cognition and that the goal of the study was to determine differential neural mechanisms, in order to minimize expectation and bias.

### Participants

Potential participants were screened using the Telephone Interview for Cognitive Status (TICS; Brandt et al., 1988) to determine preliminary eligibility. Inclusion criteria were 65–80 years of age; right-handedness; ability to speak and read English; stable medication usage for at least 30 days; willingness to complete 40 min of homework per day during the 8-week program, motivation to attend all eight classes, presence in the area and availability during the follow-up testing periods. Exclusion criteria included: any non-MRI compatible metal in body; uncontrolled high blood pressure; any cardiovascular disease; a past stroke, congestive heart failure (subjects with well-controlled vascular risk factors, such as treated hypertension or treated hyperlipidemia were included, as were subjects with a history of cerebrovascular problems but no persistent neurological deficits); uncontrolled diabetes or insulin-treated diabetes [well-controlled Type II diabetes (glucose levels <250) were included]; active hematological, renal, pulmonary, endocrine, or hepatic disorders; history of neurological disease or injury, including a history of seizures or significant head trauma (i.e., extended loss of consciousness, bleeding in the brain, Parkinson’s disease, stroke); received treatment for cancer within the last 2 years; diagnosis of schizophrenia, posttraumatic stress disorder, bipolar disorder, or psychotic disorder at any point during lifetime; any axis I psychiatric disorder within the last 12 months; any neurological or medical conditions that would interfere with study procedures or confound results, such as conditions that alter cerebral blood flow or metabolism; use of psychotropic medications or medications with CNS effects including cholinesterase inhibitors, memantine, and benzodiazepines within 12 months prior to study [medications taken on an occasional as needed basis (prn) were allowed, e.g., allergy relief]. Over the counter supplements, such as Gingko and fish oil, were also allowed; any other medications as reviewed by our team’s neurologist (BD) on a case-by-case basis. Individuals were also excluded if they engaged in current regular practice of meditation, yoga, tai chi, Feldenkrais or other mind-body practices on more than six 30-min-long sessions within the last 6 months. Any other significant prior mind-body experience was evaluated on a case-by-case basis by SL and decided upon based on frequency, duration, recency, and type of mind-body practice, with a general guideline of not more than 3 months of regular practice in the last 5 years, or more than 12 months of practice in their lifetime. Participants were also screened for physical activity levels using the Godin-Shephard Leisure-time Physical Activity questionnaire, sleep-related issues using Pittsburgh Sleep Quality Index (Buysse et al., 1989).

While familiarity with leisure activities such as crossword puzzles and sudoku, was not an exclusion criterion, participants who had prior experience with a structured cognitive fitness program such as Lumosity were excluded. Out of 74 participants that were randomized to the or Cognitive Fitness Training program, 45 had some prior experience (*n* = 27 with crossword puzzles, *n* = 15 with sudoku, *n* = 6 word jumbles, and word search, *n* = 23 with others such as solitaire, board games, or trivia games). While 10 participants had experience with two types of puzzles, none had experience with all four types trained in the course. Similarly, out of the 72 participants who were randomized to the Mindfulness Training, 28 had prior experience with yoga, tai-chi, or mantra meditation, however, the frequency of their practice was below our exclusion threshold.

Potential participants were invited to the laboratory, consented, and then underwent a structured clinical interview with our team’s neuropsychologist (BW) who performed a cognitive and functional assessment to determine final eligibility. Cognitively normal participants were determined on the basis of both an absence of cognitive symptoms and absence of impairment on cognitive testing (CDR Rating = 0; MMSE 27–30; normal performance on Trail-making Test, verbal fluency measures based on age- and education matched norms). Participants received the programs for free and were remunerated up to $275 for their participation if they completed all testing visits. Informed consent followed the guidelines of the MGH IRB.

Out of 1472 people who were screened, 146 eligible participants were found eligible and randomized into either Mindfulness Training (*n* = 72) or Cognitive Fitness Training (*n* = 74) programs. Cognitive testing and neuroimaging were conducted within a 3-week period before and after the interventions (approximately a 3-month interval). The data reported here are part of a longitudinal study with 2-year follow-up. Only baseline and post-intervention performance in our cognitive outcome measure are reported here. There was no evidence of selective attrition. Please see CONSORT diagram for additional information, including retention.

### Cognitive Outcome Measure

Our primary cognitive outcome was the Alzheimer’s Disease Cooperative Study Preclinical Alzheimer’s Cognitive Composite (PACC; Donohue et al., 2014) which consists of: (1) the Total Recall score from the Free and Cued Selective Reminding Test (FCSRT) (0–48) (Grober et al., 1988); (2) the Delayed Recall score from the Logical Memory IIa subtest from the Wechsler Memory Scale (0–25) (Wechsler, 1987); (3) the Digit Symbol Substitution Test (DSST) from the Wechsler Adult Intelligence Scale-Revised (0–93) (Wechsler, 1981); and the Mini Mental State Exam (MMSE) total score (0–30) (Folstein et al., 1975). To reduce practice effects, we administered alternate test versions at each time-point. The cognitive composite score, PACC, is determined from its components using an established normalization method (Cutter et al., 1999). Each of the four component change scores (post-pre) is divided by the baseline sample standard deviation of that component, to form standardized z scores. These z scores are summed using the following item weights as previously reported (Donohue et al., 2014): 0.72 × (FCSRT) + 0.14 × (Logical Memory IIa) + 0.12 × (MMSE) + 0.03 × (DSST). The composite score represents a standardized change score based on within-participants alterations in cognition. According to Donohue et al. (2014), the minimum treatment difference of 0.5 units is large enough to suggest a benefit to the patients and also incorporates a possible delay in later clinical deterioration.

### Cognitive Training Programs

#### Mindfulness Training Program

The Mindfulness Training (MT) program is an 8-week program that teaches mindfulness meditation exercises as a means to enhance attention and memory. The program is derived from Mindfulness-Based Stress Reduction (MBSR; Kabat-Zinn, 1990), but with an emphasis on concentration and focus rather than stress reduction. Weekly meetings lasted 1 h: 45 min meditation practice and 15 min of check-in, practice instruction, and Q&A. Participants were instructed to practice meditation at home for 45 min daily and were given guided audio recordings to facilitate practice. Weekly mindfulness instruction consisted of: Weeks 1 and 2: breath meditation and body scan; Week 3: walking meditation; Week 4: mental noting; Week 5: focus on the five physical senses and sensations; Week 6: standing meditation; Week 7: mindful eating and the five senses; Week 8: review all techniques. Participants were allowed to practice any learned technique in subsequent weeks if they desired. On average, participants attended 7.05 of eight classes and practiced 4.01 h per week at home. The program was taught by Greg Topakian, Ph.D. who has 30 years of meditation practice including 20 weeks of intensive retreat practice. He has 20 years of experience teaching in academia as well as 6 years of experience teaching secular mindfulness programs.

#### Cognitive Fitness Training Program

The Cognitive Fitness Training (CFT) program is an active control condition matched to the MT program for amount of class time and home practice. Like the MT program, class was divided into 45 min of group puzzle solving and 15 min of check-in, practice instruction, and Q&A. Weekly instruction consisted of: Week 1: word search and crossword puzzles; Weeks 2 and 3: Sudoku; Week 4: word jumbles; Weeks 5 and 6: KenKen; Weeks 7 and 8: review. Participants were given packets of puzzles to take home and instructed to practice for 45 min each day. Importantly, there was a range of difficulty available for each type of puzzle during the first week it was introduced in order to accommodate participants with different puzzle solving abilities. However, our goal was to minimize the effectiveness of this program, and so each participant continued to receive only puzzles at that chosen difficulty level for the remainder of the program, to limit development of novel strategies. On average, participants attended 6.64 of eight classes and practiced 5.99 h per week at home. The program was taught by Elisabeth Osgood-Campbell who holds a master’s degree in education and has 13 years of experience teaching in academic settings.

### MRI Data Acquisition and Analysis

#### Data Acquisition Parameters

MRI imaging was conducted in a 3T scanner (Siemens Prisma) with a 32-channel gradient head coil at the Athinoula A. Martinos Center for Biomedical Imaging in Charlestown, MA, United States. All subjects were scanned in the same scanner at both time points, i.e., within 2 weeks before (pre-scan) and within 2 weeks (post-scan) after participating in the 8-week program (∼3-month interval). We acquired T1 structural MRI images (sagittal MP-RAGE) for all subjects using the following parameters: TA = 9:14; voxel size = 1.1 mm × 1.1 mm × 1.2 mm; Rel.SNR = 1.00; slice oversampling = 0%; slices per slab = 176; TR = 2300 ms; TE = 2.01 ms; field of view = 270 mm. Subsequently, resting state functional magnetic resonance imaging (rsfMRI) were acquired using a gradient-echo echo-planar pulse sequence sensitive to the blood-oxygen-level-dependent signal (BOLD) with the following parameters: TR = 3000 ms; voxel size = 3.0 mm isotropic voxels; Rel.SNR = 1.00; interleaved slice order, slice oversampling = 0%; slice thickness = 3 mm; TE = 30 ms; Flip Angle = 85°; TA = 6:12; 46 slices, field of view = 216 mm.

#### Structural Image Processing With Voxel Based Morphometry (VBM)

Prior to preprocessing, the MP-RAGE data from 118 program participants completing both scans were visually investigated with regards to scanner artifacts as well as clinical abnormalities. After preprocessing, the scans underwent an automated quality check with the Computational Anatomy Toolbox’s (CAT12.6-rc1; v1426; Structural Brain Mapping Group, Jena, Germany) combining both measurements of noise and spatial resolution to translate into an index of weighted overall image quality. The resulting boxplot enabled a closer visual assessment of potential outliers. Moreover, the covariance between all normalized modulated images was assessed. Thereby we were able to ensure sample homogeneity.

The preprocessing for the voxel-based morphometry was conducted with CAT12’s longitudinal processing stream, which was implemented in SPM12 (Wellcome Centre for Human Neuroimaging, London, United Kingdom) running on MATLAB (R2018b) (Mathworks Inc., Natick, MA, United States). In this updated version, CAT12 is optimized to identify subtle volumetric effects resulting from training over short time periods. Default parameters were used unless specified otherwise. Individual T1-weighted MRI images for both time-points were processed by a series of steps, i.e., intra-subject alignment, bias correction, and segmentation. For the subsequent spatial normalization, we used CAT12’s template for the high-dimensional DARTEL registration with 1.5 mm (Ashburner and Friston, 2001). This approach renders a higher sensitivity for detecting regional differences (Bergouignan et al., 2009) as well as an improved normalization power because of a better inter-subject alignment (Yassa and Stark, 2009). As we were interested to investigate the actual GM values locally and to detect potential volumetric changes, images were modulated, i.e., each tissue class image was multiplied by the Jacobian determinant from the normalization matrix. Finally, images were smoothed with an 8 mm FWHM isotropic Gaussian kernel via a SPM12 standard module. Smoothed images translate into an improved normal distribution of the data, which is necessary to honor the underlying assumption for parametric statistical comparisons (Worsley et al., 1996). For the investigation of GM volume change we extracted GM values and conducted statistical analysis of our *a priori* seeds described below using SPSS version 25.

#### Seed-Based Connectivity Analyses

Resting state functional connectivity analyses were performed using the CONN toolbox v.18b (Whitfield-Gabrieli and Nieto-Castanon, 2012). Preprocessing consisted of realignment and unwarping of functional images, slice timing correction and motion correction. The functional images were resliced using a voxel size of 2 mm × 2 mm × 2 mm and smoothed using an 8-mm FWHM isotropic Gaussian kernel. ART was used detect frames with fluctuations in global signal and motion outliers. Intermediate level thresholds, which were set to reject 3% of the normative sample data, were used. The frames with motion outliers that exceeded 0.9 mm or fluctuations in global signal >5 standard were considered outliers. To address the confounding effects of participant movement and physiological noise the CompCor method (Behzadi et al., 2007) was used. The structural images were segmented into cerebrospinal fluid (CSF), white matter (WM), and GM. The principal components related to the segmented CSF and WM were extracted and were included as confound regressors in a first-level analysis along with movement parameters. The data were linearly detrended and band-pass filtered to 0.008–0.09 Hz, without regressing the global signal. Quality assessment included inspection of the sample in terms of maximum inter-scan motion, number of valid scans per subject, and scan-to-scan change in global BOLD signal and removal of outliers based on the aforementioned criteria (*n* = 20).

For the determination of seeds, an initial seed located at the posteromedial cortex seed/or in posterior cingulate/retrosplenial cortex (MNI coordinates *x* = −1, *y* = −52, *z* = 26) with an 8 mm radius was selected based on previous literature that investigated large-scale networks in older adults (Andrews-Hanna et al., 2007). The hippocampal seeds were determined based on the pattern of correlations at baseline for the whole sample using posterior cingulate/retrosplenial cortex (pC/rsp) seed. After a voxel level correction at *p* < 0.001, and a cluster level at *p*-FWE < 0.05, spherical ROIs with a radius of 8 mm were defined around the following peak coordinates within the hippocampi (hippocampus/R 30 −16 −14; hippocampus/L −26 −28 −14).

In order to assess group differences in alterations in intrinsic connectivity between our *a priori* seeds, we first examined connectivity estimates between *a priori* hippocampus and posterior cingulate/retrosplenial cortex (pC/rsp) seeds at each time point. In order to further delineate within-group changes in intrinsic connectivity in relation to changes in cognition, follow-up gPPI analyses were conducted for each group. For each group, a generalized psychophysiological interaction (gPPI) analysis computed the level of changes in functional connectivity strength between hippocampal seeds (R/L) and every voxel in the brain (post-pre), covarying with changes in cognition (PACC).

### Statistical Analysis Methods for Behavioral and Neural Outcome Measures

To assess within group differences for PACC, a one-sample *t*-test was conducted for each group, where group means were compared to a mean equal to zero, indicating no change in PACC. To assess differences in PACC between mindfulness-based and cognitive fitness trainings, an independent samples *t*-test was used. The connectivity estimates reflect the change in connectivity associated with training-dependent increases in cognition. Group differences in changes in connectivity estimates between *a priori* hippocampus and posterior cingulate/retrosplenial cortex (pC/rsp) seeds were evaluated using a repeated measures ANOVA. To assess changes in hippocampal connectivity strength covarying with changes in cognition (PACC), separate gPPI models were used for the right and the left hippocampal connectivity. For each participant (within-participants level), whole brain time series data were regressed onto the ROI signal to generate connectivity maps at each time point (baseline and post-intervention). Post intervention bivariate regression coefficient maps were then subtracted from baseline maps to create a map of whole-brain connectivity changes with each hippocampal seed for each participant. At the second (between-participants) level, these change maps were then regressed onto PACC scores to create a map of regions whose connectivity change significantly correlated with PACC. To explore changes related to MT, first the gPPI analysis was run on participants from the MT group, followed by the CFT group alone. Both gPPI statistics were evaluated via SPM 8 using a voxel level threshold at *p* < 0.001, and a cluster level threshold at *p*-FWE < 0.05 for multiple comparisons. Bivariate regression coefficients were then extracted from all participants at each time-point to allow for comparison of MT changes relative to the CFT group.

## Results

### Cognitive Outcomes

**FIGURE 1:**
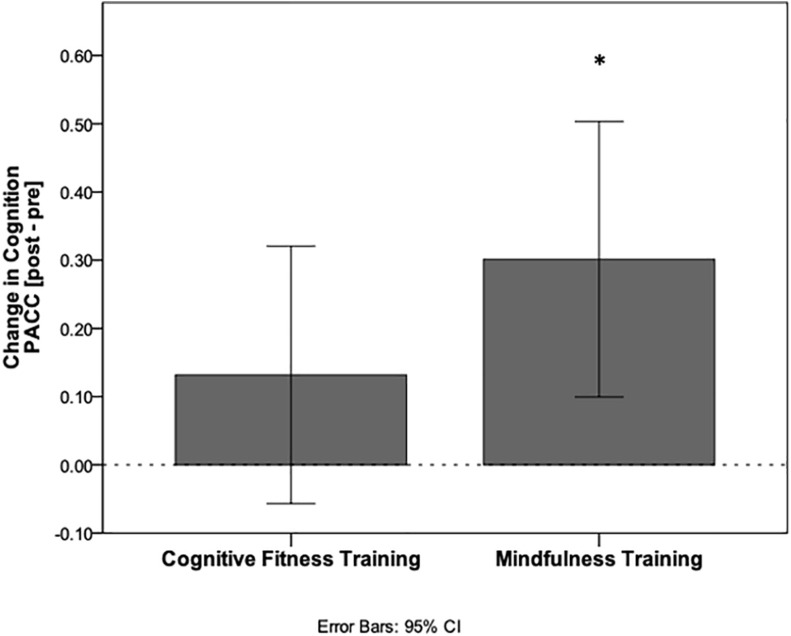
Cognitive improvement relative to baseline performance. PACC scores calculated as change from baseline following the interventions for each group. Mindfulness training resulted in a significant within-group increase in cognition (*p < 0.05), while cognitive fitness training did not.

In the MT group, PACC scores increased after the intervention compared to baseline [0.21 mean increase ± 0.68 standard deviations (SD); *t*(60) = 2.44, *p* = 0.018, CI (0.04–0.39), Cohen’s *d* 0.31]. In the CFT group, PACC scores did not increase relative to baseline following the intervention [0.10 mean increase ± 0.64 SD; *t*(64) = 1.30, *p* = 0.20, CI (−0.06 to 0.26), Cohen’s *d* 0.16]. Despite these findings, the between-group comparison was not statistically significant [*t*(124) = 0.942, *p* = 0.348, CI (−0.121, 0.342), neuroimaging sample *t*(95) = 1.235, *p* = 0.220, CI (−0.103, 0.442), Figure 1].

Baseline characteristics of the whole sample are presented in Table 1. Performance on PACC as well as performance on each cognitive test at each time point are presented in Table 2. There were no differences between groups in FCSRT [*t*(124) = 0.142, *p* = 0.888], in Logical Memory IIa [*t*(124) = −0.040, *p* = 0.968], in MMSE [*t*(124) = 0.946, *p* = 0.346], or in DSST [*t*(124) = 1.291, *p* = 0.199] at baseline. The significant improvement in the PACC composite score for the MT group was driven by primarily by an increase in the FCSRT total recall that was not seen in the control group. Both groups improved on LMIIa delayed recall performance and showed slight improvements on digit symbol substitution test. The MMSE was uninformative in this study because many participants performed at ceiling at baseline.

**TABLE 1:** Baseline characteristics of study participants.

	Mindfulness training	Cognitive fitness training	Statistical test value	*p*	Cohen’s *d*
Sample size	70	75			
Age (years)	70.2 ± 4.1 (*n* = 70)	71.0 ± 4.3 (*n* = 75)	*t* = −1.10	0.27	0.19
Gender (% female)	55.7 (*n* = 70)	53.3 (*n* = 75)	χ(1) = 0.08	0.77	
Education (years)	16.7 ± 1.8 (*n* = 69)	16.7 ± 1.9 (*n* = 74)	*t* = 0.12	0.91	0.00
Education (ISCED level)	6.5 ± 1.0 (*n* = 69)	6.5 ± 1.1 (*n* = 74)	*t* = 0.10	0.92	0.00

*Numbers denote mean ± standard deviation. *p* indicates the significance of the group differences on Students’ *t* or chi-square test.*

**TABLE 2:** Cognitive outcome measures.

Mindfulness training					
	
	Pre	Post	*t*	*p*	Cohen’s *d*
**PACC**	*n* = 70 (*n* = 61)	*n* = 61			
Digit symbol	51.8 ± 9.7 (51.3 ± 9.3)	52.6 ± 9.1	−1.66	0.10	0.09
MMSE	29.1 ± 1.0 (26.1 ± 1.1)	29.0 ± 1.3	0.25	0.80	0.09
FCSRT total recall	30.8 ± 5.8 (31.2 ± 5.7)	32.3 ± 4.8	−1.93	0.06	0.28
LMIIa delayed recall	12.2 ± 4.2 (12.3 ± 4.2)	13.9 ± 4.1	−2.70	0.009	0.41
**Total PACC change (*n* = 61)**	**0.21 ± 0.68**				

**Cognitive fitness training**					
	
	**Pre**	**Post**	***t***	***p***	**Cohen’s *d***

**PACC**	*n* = 75 (*n* = 65)	*n* = 65			
Digit symbol	49.0 ± 11.0 (48.9 ± 11.6)	50.3 ± 11.1	−1.83	0.07	0.12
MMSE	29.0 ± 1.0 (28.9 ± 1.0)	28.8 ± 1.0	1.14	0.26	0.2
Free recall	31.1 ± 5.1 (31.1 ± 5.2)	31.4 ± 4.9	−0.35	0.73	0.06
Delayed recall	12.5 ± 3.3 (12.3 ± 3.3)	14.7 ± 4.0	−5.12	<0.001	0.6
**Total PACC change (*n* = 65)**	**0.10 ± 0.64**				

*Numbers denote mean ± standard deviation for each cognitive measure, and PACC; *p* values pertain to paired *t*-tests between participants with both baseline and follow-up measures.*

There was no significant difference between groups in terms of their physical activity [*t*(130) = 0.890, *p* = 0.375], or sleep levels [*t*(124) = 0.468, *p* = 0.641] at baseline either. The changes in PSQI scores from baseline to post-testing did not differ between groups [*F*(1,126) = 1.194, *p* = 0.277, η^2^ = 0.01]. Mindfulness Training group had the following PSQI scores at baseline (4.83 ± 3.03), and at post (4.66 ± 2.92), while the Cognitive Fitness Training group had the following PSQI scores at baseline (4.72 ± 3.06), and at post (4.75 ± 2.95). The changes in exercise scores from baseline to post-testing did not differ between groups either [*F*(1,114) = 2.748, *p* = 0.100, η^2^ = 0.24]. Mindfulness Training group had the following Godin exercise scores at baseline (34.89 ± 20.10), and at post (35.75 ± 2157), while the Cognitive Fitness Training group had the following scores at baseline (38.64 ± 27.58), and at post (49.72 ± 35.73).

### Mindfulness Training Is Associated With Increased Intrinsic Connectivity Between the Right Hippocampus and Posteromedial Cortex

To assess group differences in alterations in intrinsic connectivity between our *a priori* seeds, we first examined connectivity estimates between *a priori* hippocampus and posterior cingulate/retrosplenial cortex (pC/rsp) seeds at each time point. An investigation of group differences in changes connectivity estimates between pC/rsp and left hippocampus seed [*F*(1,95) = 0.048, *p* = 0.827, η^2^ = 0.001], and between pC/rsp and right hippocampus seed [*F*(1,95) = 0.011, *p* = 0.916, η^2^ = 0.000] did not reveal any differences between groups over time.

**FIGURE 2:**
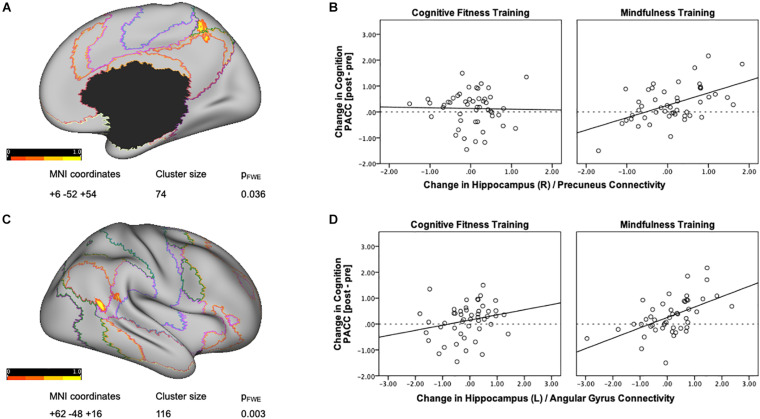
Training-dependent changes in hippocampal connectivity strength that covary with changes in cognition. **(A)** A whole brain gPPI analysis of changes in functional connectivity (baseline vs. post-intervention) using change in PACC scores as regressor and right hippocampus as seed resulted in a significant cluster at the right precuneus for the mindfulness training group. Networks are based on the Yeo seven-network parcellation (Yeo et al., 2011) and are represented by the following colors: violet: visual, blue: somato-motor, green: dorsal attention, pink: ventral attention, cream: limbic, orange: fronto-parietal, and red: default network. **(B)** Mindfulness-training-dependent improvements in PACC cognitive composite scores correlated with increases in intrinsic connectivity between the right hippocampus and the right precuneus, while the Cognitive Fitness Training group showed no association. The connectivity estimates reflect the change in connectivity strength associated with training-dependent increases in cognition, and were plotted using SPSS v.24 (Chart Editor). The fitted regression line reflects the best estimate of the connectivity between the hippocampus and the precuneus in **B**. **(C)** A whole brain gPPI analysis of the changes in functional connectivity (baseline vs. post-intervention) using change in PACC scores as regressor and left hippocampus as seed resulted in a significant cluster at the right angular gyrus for the mindfulness training group. Networks are based on the Yeo seven-network parcellation (Yeo et al., 2011) and are represented by the following colors: violet: visual, blue: somato-motor, green: dorsal attention, pink: ventral attention, cream: limbic, orange: fronto-parietal, and red: default network. **(D)** Mindfulness-training-dependent improvements in cognitive composite scores correlated with increases in intrinsic connectivity between the left hippocampus and the right angular gyrus, while the Cognitive Fitness Training group showed no association. The connectivity estimates reflect the change in connectivity strength associated with training-dependent increases in cognition, and were plotted using SPSS v.24 (Chart Editor). The fitted regression line reflects the best estimate of the connectivity between the left hippocampus and the right angular gyrus in **C**.

Next, in order to delineate mindfulness training dependent changes in hippocampal connectivity strength that covary with changes in cognition, we conducted a whole brain gPPI analysis (baseline vs. post-intervention) using PACC scores as regressor and right hippocampus as seed region. This analysis resulted in a significant cluster at right precuneus for the mindfulness training group [MNI coordinates +6 −52 +54, cluster size (*k*) = 74, *p*-FWE = 0.037, Figure 2A]. Mindfulness-training dependent improvements in cognitive composite scores were associated with increases in intrinsic connectivity between the right hippocampus and right precuneus (*r* = 0.526, *p* < 0.001, Figure 2B). A parallel whole brain gPPI analysis (baseline vs. post-intervention) using PACC scores as regressor and right hippocampus as seed region in the CFT group did not yield any results. In order to compare two groups, connectivity estimates between the right hippocampus and the cluster in the precuneus were extracted. While there was no association between improvements in cognitive composite scores and increases in intrinsic connectivity between the right hippocampus and right precuneus in the CFT group (*r* = −0.023, *p* = 0.876, Figure 2B), a test for between-group differences was not significant [*F*(1,95) = 0.264, *p* = 0.609, η^2^ = 0.003].

### Mindfulness Training Is Associated With Increased Intrinsic Connectivity Between the Left Hippocampus and the Right Angular Gyrus

A whole brain gPPI analysis (baseline vs. post-intervention) using PACC scores as regressor and left hippocampus as seed region resulted in a significant cluster in the right angular gyrus for the mindfulness training group [MNI coordinates +62 −48 +16, cluster size (*k*) = 116, *p*-FWE = 0.003, Figure 2C]. Mindfulness-training dependent improvements in cognitive composite scores were associated with increases in intrinsic connectivity between the left hippocampus and the right angular gyrus (*r* = 0.538, *p* = 0.000, Figure 2D). A parallel whole brain gPPI analysis (baseline vs. post-intervention) using PACC scores as regressor and left hippocampus as seed region in the CFT group did not yield any results. In order to compare two groups, connectivity estimates between the left hippocampus and angular gyrus were extracted as well. While there was no association between improvements in cognitive composite scores and increases in intrinsic connectivity between the left hippocampus and angular gyrus (*r* = 0.232, *p* = 0.108, Figure 2D) for the CFT group, and a test for between-group differences was not significant [*F*(1,95) = 1.647, *p* = 0.203, η^2^ = 0.017].

Our hypotheses about changes in gray matter volume were not supported. There was no main effect of time nor any significant within-group changes within our ROIs for the mindfulness group (all *p* > 0.48). There was a main effect of time in the right hippocampus for the CFT group which did not survive multiple comparisons correction. Moreover, in opposition to our *a priori* hypothesis, we were not able to identify any significant results when correlating GMV change values with PACC change scores.

## Discussion

In the present study, we performed a randomized controlled trial to test the hypothesis that mindfulness training can maintain or improve cognitive function in healthy older adults, and we used functional and structural MRI to investigate the neural basis of cognitive outcome. We found that an 8-week mindfulness-based training program improved cognition as assessed by Preclinical Alzheimer’s Cognitive Composite (PACC) in cognitively normal older adults, and that these improvements were associated with increased intrinsic connectivity within the default mode network, particularly between the right hippocampus and precuneus and between the left hippocampus and right lateral parietal cortex. Although the active control group did not show these effects, we were not able to demonstrate a statistically significant between-group difference in the primary cognitive outcome measure, likely because the effect size of the mindfulness program was small over this relatively short period of time, control training program was more active than anticipated and/or due to overlaps between the two programs in terms of their utilization of attention and attentional control mechanisms. Nevertheless, these findings suggest that additional longer-term studies of the potential benefits of mindfulness training should be investigated as an activity that could potentially contribute to the prevention of age-related cognitive decline.

The enhanced cognition scores following mindfulness training can be attributed primarily to improved episodic memory performance on both the Free and Cued Selective Reminding Test and the Logical Memory II Delayed Recall Test (Wechsler, 1987; Grober et al., 2008). These findings are consistent with several reviews and meta-analyses which reported moderate effects of mindfulness training on memory specificity (Chiesa et al., 2011; Gard et al., 2014; Lao et al., 2016; Fountain-Zaragoza and Prakash, 2017). The “brain games” practiced by the control group included crossword puzzles and word jumbles, both of which engage semantic memory (Pillai et al., 2011). Thus the lack of between group differences is likely due to the fact that engaging in meaningful mental stimulation and intellectual activity can improve performance on tasks that tap into the same cognitive domain that is trained (Aguirre et al., 2013). Importantly, while neither group exhibited significant levels of improvement in free recall, while the mindfulness training exhibited an improvement that approached significance. Here it is important to note the sensitivity of episodic memory to age-related decline (Donohue et al., 2014). Therefore, an improvement in this ability may be deemed to have potential clinical significance, especially in delaying age-dependent memory decline. Compared to other training programs in healthy older adults that found little to no improvements in memory (Gross et al., 2012), current findings of training-dependent improvements in the PACC, particularly in free recall, further support the use of mindfulness training as an activity to promote successful cognitive aging.

Growing evidence suggests that age-related cognitive decline is associated with changes in functional connectivity within and between large-scale brain networks (Andrews-Hanna et al., 2007; Ferreira and Busatto, 2013; Damoiseaux, 2017). Relative to younger adults, cognitively intact older adults show reduced functional connectivity within the default mode network at rest (Ward et al., 2015; Damoiseaux, 2017; Staffaroni et al., 2018), as well as less pronounced deactivations during cognitive tasks (Grady et al., 2006; Persson et al., 2014; Spreng et al., 2016). Decreased resting state connectivity between the hippocampus and precuneus/posterior cingulate have been implicated in typical age-related cognitive decline (Wang et al., 2010; Bernard et al., 2015; Li et al., 2020). Although studies with cross-sectional populations suggest that there is a low share for the overall connectivity strength of the default network in explaining the age-related variance across various cognitive domains (Hedden et al., 2016), here, in a large sample of cognitively normal older adults, we report an association between improved cognition and training-dependent increases in the intrinsic connectivity between the right hippocampus and precuneus and between the left hippocampus and right lateral parietal cortex.

The increase in functional connectivity between the posteromedial cortex and hippocampus in the mindfulness training group was strongly associated with increases in episodic memory. This finding supports our initial hypothesis that mindfulness training may improve cognition in part through changed connectivity within the default mode network. Such an interpretation is also congruent with reports of a hippocampal-parietal network that is associated with episodic memory retrieval (Vincent et al., 2006), reports of positive association between default network connectivity and episodic memory (Huo et al., 2018), reports of an association between greater within network functional connectivity and cognitive status in healthy older adults (Sullivan et al., 2019), as well as with reports of mindfulness training dependent connectivity increases within the default mode network (Brewer et al., 2011; Taylor et al., 2013; Wells et al., 2013). The posterior parietal cortex and the precuneus are among the regions most frequently activated during both successful memory formation and episodic memory retrieval (Buckner et al., 2008; Spreng et al., 2009), and are particularly susceptible to neuropathological changes associated with aging and Alzheimer’s Dementia (Buckner, 2004). Thus, present findings help substantiate the idea that enhanced intrinsic connectivity between the hippocampus and posteromedial cortex may represent one neural mechanism by which mindfulness training promotes memory function in healthy older adults.

We also identified a mindfulness training related increase in coordinated neural activity between left hippocampus and right angular gyrus. This finding is in accordance with our prior findings of mindfulness training dependent reorganization of hippocampal-cortical networks during retrieval of extinguished fear memories (Sevinc et al., 2019, 2020). Both the precuneus and the angular gyrus are part of the dorsal medial subsystem of the default mode network, that have been associated with metacognitive reflection (D’Argembeau et al., 2014). The dorsomedial and the medial temporal subsystems are closely linked, and both have been shown to be recruited during memory tasks (Andrews-Hanna et al., 2010). Critically, the angular gyrus is part of the ventral parietal cortex that is thought to direct attention to memory contents (Cabeza et al., 2008; Ciaramelli et al., 2008). Although future task-based studies are needed, the results suggest that mindfulness-training based increases in the ability to direct attention to memory contents may be one of the mechanisms through which mindfulness training increases memory performance.

While the design of the present study precludes determining whether the observed memory enhancements resulted from improved encoding or retrieval, consistent with research documenting the relation between mindfulness and attention, we had originally hypothesized that mindfulness training-dependent enhanced awareness of present moment experience would contribute to memory encoding (see Chiesa et al., 2011; Tang et al., 2015, for reviews). The present data suggest that mindfulness training related enhanced connectivity within the default mode network may contribute to improved memory via enhanced encoding or enhanced retrieval mechanisms. These findings are also in agreement with reports of meditation practice moderating aging-related decrements in measures of sustained attention (Zanesco et al., 2018). Conducting more nuanced memory tasks within the MRI scanner will be required to precisely define the impact of mindfulness training on each component of memory encoding and retrieval.

The PACC cognitive composite utilized in the study has been designed to be sensitive to cognitive changes in older adults, especially to the earliest signs of cognitive decline in Alzheimer’s disease (AD; Donohue et al., 2014). Test scores that constitute the composite scores have long been used as primary markers of disease progression as well as measure of treatment effects (Amieva et al., 2008, 2019). PACC performance has reliably characterized and quantified the risk for Alzheimer-related cognitive decline among cognitively normal individuals with elevated levels of brain amyloid (Donohue et al., 2017). Consequently, a low score on the Free Recall measure has been suggested as a core neuropsychological marker of prodromal AD (Auriacombe et al., 2010). Similarly, alterations in connectivity between the precuneus/posterior cingulate and the hippocampus during rest have been implicated in MCI and AD patients (Wang et al., 2006; Sperling et al., 2010; Çiftçi, 2011; Vannini et al., 2013). Thus, training dependent increases in precuneus-hippocampal connectivity seen in the current study suggest that mindfulness-training may also be one of the mechanisms through which mindfulness training improves memory in individuals with mild cognitive impairment (Wells et al., 2013; Yang et al., 2016; Wong et al., 2017), and also contribute to discussions around brain regions associated with cognitive reserve in aging (Solé-Padullés et al., 2009).

An important strength of the study was the use of a “stripped down” mindfulness program which focused exclusively on teaching formal mindfulness meditation exercises and did not contain any psycho-education, or cognitive or behavioral therapy elements. Further, we used an engaging, credible, active control condition which was portrayed to the participants as being equally efficacious as the mindfulness program. Together, these study design elements allowed us to identify effects that were specifically attributable to mindfulness practice rather than to generic effects of participating in a group activity, or to other therapeutic elements that are usually included in clinical mindfulness based interventions (Kabat-Zinn, 1990; Segal and Williams, 2002). As they were no differences between groups in terms of changes in physical activity or sleep quality over time, it is unlikely that the reported changes are due to these potential mediators. Other strengths of the study include the large sample size, blinded outcome of assessors, highly experienced teachers, and excellent participant compliance and retention, all of which have been major issues for many prior mindfulness studies (Chiesa et al., 2011; Tang et al., 2015; van der Velden et al., 2015; Lao et al., 2016; Van Dam et al., 2018).

The primary limitation of the study is that despite our efforts to match the groups on amount of time spent practicing at home, the CFT group practiced considerably more than what was prescribed, while the MT group practiced slightly less than prescribed. Therefore, this study bears the risks of type II errors, i.e., omitting potential group-by-time effects undermined by differential adherence to the study design. However, the within-group analyses help circumvent this issue, as do the differential correlations between brain and cognitive changes. Furthermore, some of the participants in the CFT group were already familiar with the training materials used in the program, which could contribute to smaller effect sizes in the CFT group. Thus, null training effect in the CFT group may be partially explained by their familiarity with some of the training materials prior to enrollment. As such, major limitation of the study is the lack of a significant between-group difference. Future research is needed to assess dissociable cognitive outcomes using more specified attentional measures and associated neural mechanisms of action. Future research may also assess whether the neural changes and cognitive improvements reported in this study are affected from confounding factors such as age, sex, education, whether these gains also translate into tangible gains in everyday life activities, whether the positive effects observed will be maintained over a longer period of time, and to what degree these interventions can delay the onset of various forms of cognitive decline.

## Data Availability Statement

The raw data supporting the conclusions of this article will be made available by the authors, without undue reservation.

## Ethics Statement

The studies involving human participants were reviewed and approved by the Mass General Brigham Human Research Committee. The patients/participants provided their written informed consent to participate in this study.

## Author Contributions

SL and BD contributed to the conception and design of the study. GS, TD, RK, SG, MS, and NT carried out the data collection. GS and JR performed the statistical analysis and wrote the first draft of the manuscript. SL oversaw all the data collection and analysis. CG, GT, DR, and BD oversaw the data analysis. DR, BD, and SL contributed to the interpretation of the results. All authors provided critical feedback, discussed the results, and commented on and approved the submitted manuscript.

## Conflict of Interest

The authors declare that the research was conducted in the absence of any commercial or financial relationships that could be construed as a potential conflict of interest.

## Publisher’s Note

All claims expressed in this article are solely those of the authors and do not necessarily represent those of their affiliated organizations, or those of the publisher, the editors and the reviewers. Any product that may be evaluated in this article, or claim that may be made by its manufacturer, is not guaranteed or endorsed by the publisher.
