# Supplementary figures and images for: Mindfulness Training Improves Cognition and Strengthens Intrinsic Connectivity Between the Hippocampus and Posteromedial Cortex in Healthy Older Adults
Source: Front Aging Neurosci. 2021 Aug 27;13:702796. doi: 10.3389/fnagi.2021.702796 (PMC8430251; doi:10.3389/fnagi.2021.702796)

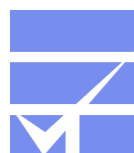

# CONSORT

TRANSPARENT REPORTING of TRIALS

## CONSORT 2010 Flow Diagram

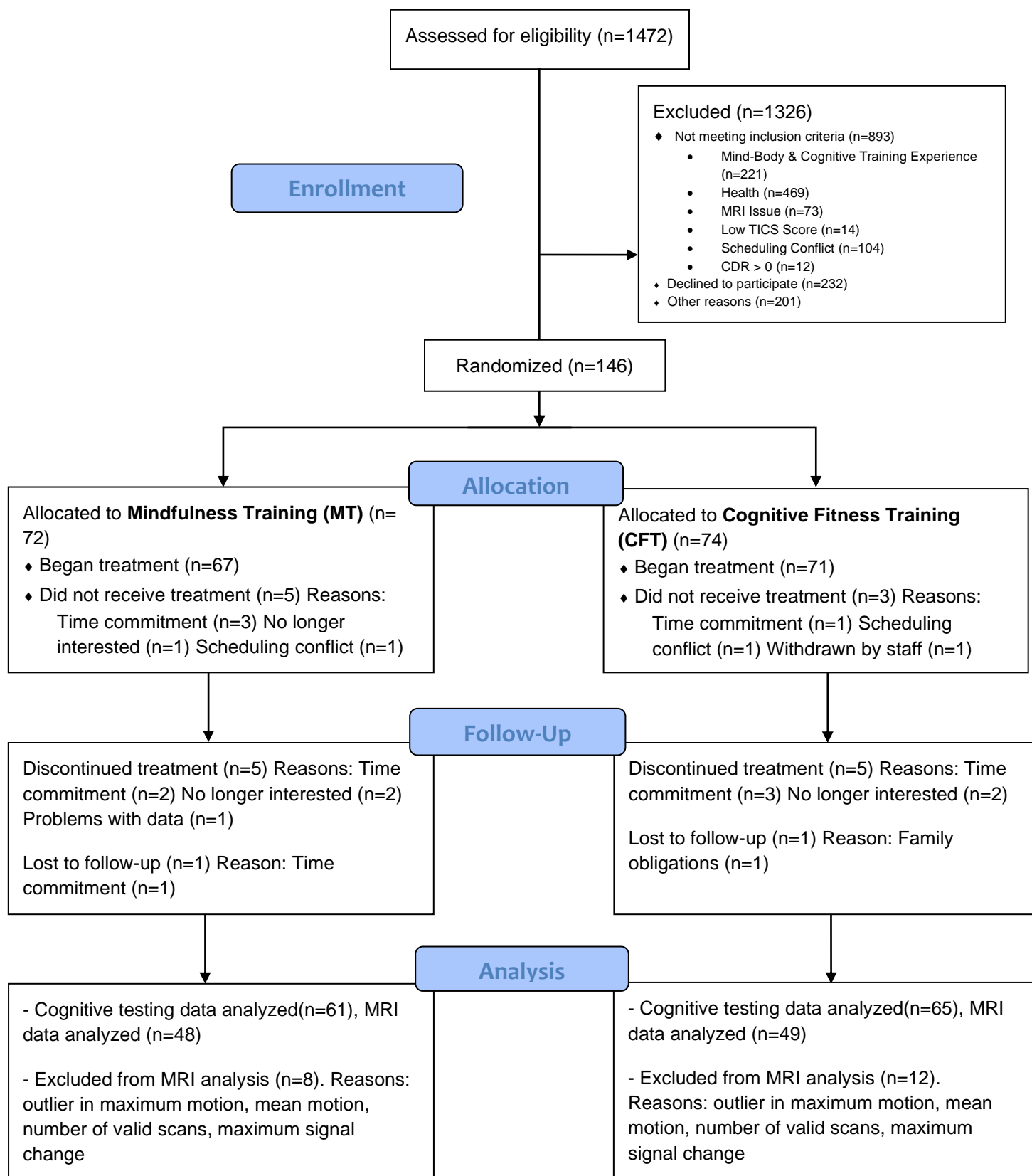

Supplement: Supplementary file 1 [file Data_Sheet_1.pdf]
